# Converting Potential Abdominal Hysterectomy to Vaginal One: Laparoscopic Assisted Vaginal Hysterectomy

**DOI:** 10.1155/2014/305614

**Published:** 2014-03-05

**Authors:** Jyothi Shetty, Asha Shanbhag, Deeksha Pandey

**Affiliations:** ^1^KMC, Manipal University, Manipal 576104, India; ^2^KMC, Manipal University, Mangalore 575001, India

## Abstract

*Background.* The idea of laparoscopic assisted vaginal hysterectomy (LAVH) is to convert a potential abdominal hysterectomy to a vaginal one, thus decreasing associated morbidity and hastening recovery. We compared intraoperative and postoperative outcomes between LAVH and abdominal hysterectomy, to find out if LAVH achieves better clinical results compared with abdominal hysterectomy. *Material and methods.* A total of 48 women were enrolled in the study. Finally 17 patients underwent LAVH (cases) and 20 underwent abdominal hysterectomy (controls). All surgeries were performed by a set of gynecologists with more or less same level of surgical experience and expertise. *Results.*None of the patients in LAVH required conversion to laparotomy. Mean operating time was 30 minutes longer in LAVH group as compared to abdominal hysterectomy group (167.06 + 31.97 min versus 135.25 + 31.72 min; *P* < 0.05). However, the mean blood loss in LAVH was 100 mL lesser than that in abdominal hysterectomy and the difference was found to be statistically significant (248.24 + 117.79 mL versus 340.00 + 119.86 mL; *P* < 0.05). Another advantage of LAVH was significantly lower pain scores on second and third postoperative days. Overall complications and postoperative hospital stay were not significantly different between the two groups.

## 1. Background

Hysterectomy is the second most frequently performed major surgical procedure on women all over the world, next only to cesarean. The term “hysterectomy” though means removal of uterus; in practice it has a much wider classification depending upon the indication. At times, it is done without removal of the cervix (supracervical hysterectomy) or with removal of adnexa (hysterectomy with salpingooophorectomy). It can also be a part of staging laparotomy or radical hysterectomy. Hysterectomy can be performed abdominally, vaginally, or through abdominal ports with help of laparoscope. Approach depends on surgeon's preference, indication for surgery, nature of the disease, and patient characteristics. As any other surgery, hysterectomy is also associated with intraoperative and postoperative complications. Rates of various complications with hysterectomy have been reported in the range of 0.5% to 43% [1].

There is enough evidence from multiple randomized trials that vaginal hysterectomy is associated with fewer complications, a shorter hospital stay, more rapid recovery, and lower overall cost [2]. The idea of laparoscopic assisted vaginal hysterectomy (LAVH) is to convert a potential abdominal hysterectomy to a vaginal one, thus decreasing associated morbidity and hastening recovery. LAVH after being reported for the first time in 1989 gained wide popularity within a decade or two. Johnson et al. found that LAVH decreased pain, surgical site infections, and hospital stay and led to a quicker return to normal activities and fewer postoperative adhesions [3]. Quality of life studies also proved it to be better than abdominal hysterectomy at six weeks postoperatively [4]. However, Sculpher et al. could not demonstrate that LAVH was better than abdominal hysterectomy in their circumstances. The more we go through the literature and compare more variables among the two approaches, it is realized that the question of LAVH versus abdominal hysterectomy becomes more and more confusing [5].

Thus in this prospective study we aimed to compare the intraoperative and postoperative outcome between LAVH and abdominal hysterectomy, in order to find out if LAVH achieves better clinical results compared with abdominal hysterectomy.

## 2. Material and Methods

The present study was a prospective comparative study performed in a university teaching hospital from October 2007 to July 2009. The study was approved by the institutional ethical review board. Our study population was recruited from the set of women who were admitted in our hospital and required hysterectomy for the management of benign gynecological conditions. In order to convert a potential abdominal hysterectomy to a vaginal one with the help of LAVH we included those women who either had concomitant adnexal mass requiring adnexectomy, women who had undergone previous abdominopelvic surgery (like myomectomy, hysterotomy, surgeries on adnexa, and cesarean deliveries; and might require adhesiolysis), or women with history of pelvic inflammatory disease (PID) or endometriosis with suspected adhesions. Patients with one or more contraindications to LAVH were excluded from the study. This included cardiac or respiratory morbidity, frozen pelvis, broad ligament fibroid, and cervix flushed with vagina.

After recruiting the patients, they were informed about the study and written consent was obtained.

Women with benign gynecological conditions who required hysterectomy and where vaginal hysterectomy was not an option were recruited for the study. All these women were explained in detail about the advantages (abdominal hysterectomy: less operating time, regional anaesthesia, less cost; LAVH: less pain, cosmetic benefit) and disadvantages (abdominal hysterectomy: bigger incision, more postoperative pain; LAVH: chance of conversion to open method, only option of general anaesthesia, more time) of both the procedures with the help of a pre-prepared information leaflet which was based on the literature review. Patients were then allowed to choose from the two methods. A written consent was obtained from all the participants. All patients were given an oral gut lavage solution containing polyethylene glycol, sodium chloride, potassium chloride, and sodium bicarbonate, 1.5 liters ingested over 2-3 hours. Proctoclysis enema was administered the night before and also in the morning of the day of surgery. Patients were kept nil per oral for 12 hours before the surgery. Antiseptic vaginal douche was done preoperatively. All patients were subjected to prophylactic intravenous antibiotic half an hour before surgery and then eighth hourly in the postoperative period for 48 hours (amoxicillin 1000 mg + clavulanic acid 200 mg). Additional antibiotic was added if the same was deemed necessary due to any postoperative infection. General anesthesia was administered to all patients.

All surgeries were performed by a set of gynecologists with more or less same level of surgical experience and expertise. Abdominal hysterectomy was performed by the extrafascial technique and the vaginal cuff was sutured with interrupted sutures. LAVH was performed using video monitoring equipment. A 10 mm laparoscope with a Storz endovision camera was inserted in a subumbilical position. Three more 5 mm entry ports were created, one on each right and left spinoumbilical line and one on midline suprapubic region 3 cm above the symphysis pubis. Opening of bladder flap was done laparoscopically whereas bladder dissection was done during the vaginal phase of hysterectomy. Vaginal phase of hysterectomy was commenced with an anterior circumferential incision of the vagina. At the end after closing the vaginal cuff, a pneumoperitoneum was recreated to confirm hemostasis. A decision to convert a LAVH to an abdominal hysterectomy was readily made if difficulties were encountered. Following both, Foleys urinary catheter was left in situ and was removed after 24 hours or later depending upon the individual case. In LAVH, a vaginal pack was left in situ which was also removed 24 hours later. Postoperatively, all patients were prescribed an identical regimen of analgesia. A diclofenac rectal suppository was initially administered at the time of completion of the surgery. Following this, intramuscular tramadol and diclofenac rectal suppository were administered twice a day on the first postoperative day and then according to the patient's request.

### 2.1. Outcome Measures

The duration of surgery was calculated from the first surgical incision to the time when the last skin suture was applied. Blood loss during the laparoscopic phase was calculated as the difference between the volume of fluid aspirated and that of the fluid introduced into the pelvic cavity. Blood loss during the vaginal phase of LAVH or during abdominal hysterectomy was determined directly from the aspirated fluid collected in the calibrated container. Sponges used for mopping were also taken into consideration and one fully socked sponge was accounted for 50 mL of blood loss. All intraoperative complications—damage to ureter, urinary bladder, or major vessels and torrential hemorrhage requiring either transfusion or conversion of LAVH to open method—were noted. The specimen weight was obtained immediately after the surgery. Hemoglobin estimation was done for all patients 24 hours after surgery and blood transfusion was given if the hemoglobin was less than 8 gm/dL. Postoperative fever was considered as body temperature of more than 38.2°C for two consecutive measurements at least 6 hours apart, excluding the first 24 hours following the surgery. For comparing postoperative pain, we used visual analogue scale (VAS) in our study. Other postoperative complications like wound infection, secondary hemorrhage, or pulmonary embolism were also noted. For calculation of hospital stay, only days from surgery till discharge from the hospital were taken into account. The patients were discharged once they were able to tolerate oral diet, could void normally, were ambulatory, did not require parenteral medication, and had stable hematocrit.

### 2.2. Statistical Analysis

Statistical Package for the Social Sciences (SPSS 11.5 for Windows) was used for data compilation and statistical analysis. Independent sample *t*-test was used for discrete and continuous variables. Independent *t*-test was applied to test the difference between mean values of the variables in the two groups compared. Mann-Whitney test was used when variables had a nonparametric distribution (to compare number of previous surgeries, intraoperative blood loss, and weight of the retrieved specimen). Chi-square test was applied to those tests that evaluate the possible effect of one variable upon an outcome (postoperative complications). Fischer's exact test was used to compare the rate of postoperative wound infection, as the frequency was less than five.

## 3. Results

A total of 48 women were enrolled in the study. Out of these only 37 could be included as two were found to have frozen pelvis, one had broad ligament fibroid, and four menopausal women were found to have cervix flushed with vagina. In the remaining four women, their cardiorespiratory status contraindicated laparoscopy. Finally, 17 patients underwent LAVH (cases) and 20 underwent abdominal hysterectomy (controls). None of the patients in LAVH required conversion to laparotomy.

Demographic characteristics of both the groups have been tabulated in Table 1. Mean age of women in the LAVH group was 43.2 years as compared to 49.8 years in the abdominal hysterectomy group. Other characteristics like parity, cesarean deliveries, previous pelvic surgeries, and body mass index (BMI) were also comparable in both the groups. Even the comorbidities such as hypertension, diabetes mellitus, and thyroid disorders were also equally distributed between the two groups. Majority of women in both groups underwent hysterectomy for symptomatic fibroid uterus (58.8% in LAVH group and 45% in abdominal hysterectomy group), the next common indication being dysfunctional uterine bleeding (DUB) (23.5% in LAVH group and 25% in abdominal hysterectomy group).

It was observed that the mean operating time for LAVH was 30 minutes longer than that for abdominal hysterectomy and this was statistically significant (167.06 ± 31.97 min versus 135.25 ± 31.72 min; *P* < 0.05). However the mean blood loss in LAVH was 100 mL lesser than that in abdominal hysterectomy and the difference was found to be statistically significant (248.24 ± 117.79 mL versus 340.00 ± 119.86 mL; *P* < 0.05). Four patients in the abdominal hysterectomy group required packed cell transfusion in the postoperative period while none of the patients required transfusion in the intraoperative or postoperative period. Weight of the uteri removed in both the groups was found to be comparable (223.82 ± 71.6 g in LAVH versus 252.00 ± 151.92 g in abdominal hysterectomy) (Table 2).

**Figure 1:**
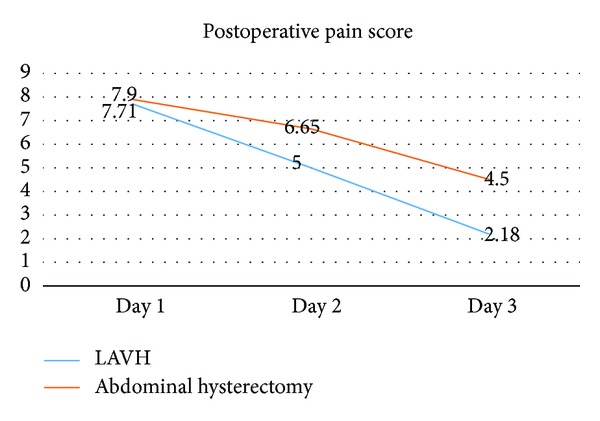
Pain scores among the two methods following surgery.

Six patients in the abdominal hysterectomy group required extra analgesia in the first postoperative day as compared to none in the LAVH group. The measurement of pain perception in the postoperative period was done with the help of VAS, where patients rated 10 for excruciating pain and 0 for no pain. It was seen that the level of pain (represented as mean ± standard deviation), perceived on the second and third postoperative days was significantly lower in the LAVH group. Difference of pain scores was not significant among the two procedures at Day 1 (Figure 1).

Table 3 shows the rate of postoperative complications in both the groups. In our study, the complications were more or less similar in both the groups. Even the postoperative hospital stay was also not significantly different in both the groups.

## 4. Discussion

In our study, it was found that the mean operating time was 30 minutes longer in LAVH group as compared to abdominal hysterectomy group. However the mean estimated blood loss was around 90 mL more in abdominal hysterectomy group. Another advantage of LAVH was significantly lower pain scores on second and third postoperative days. Overall complications were not significantly different between the two groups.

In a study conducted by Summitt et al. also the operative time averaged 30 minutes longer in LAVH and estimated blood loss averaged 100 mL greater in abdominal hysterectomy with no difference in the rate of overall complications [6]. While in another study by Marana et al. there was no difference between the operating time between the two groups. However, the estimated blood loss was around 89 mL higher in the abdominal hysterectomy group [7]. Similar to our observation, Marana et al. also found a significant reduced pain perception on second and third postoperative days in patients who underwent LAVH [7].

A recent meta-analysis which compared 23 randomized controlled trials concluded that LAVH has a significantly longer operation time than abdominal hysterectomy. This may be due to the learning curve for laparoscopy requiring a high level of skill and good hand-eye coordination. However, we also agree with the authors (Yi et al) that comprehensive training of surgeons and the development of surgical instruments may lead to a decrease in the operation time for LAVH in the future. Similar to our results in the meta-analysis also postoperative pain and hemoglobin drop were reduced significantly, and return to normal activities was significantly quicker following LAVH compared with abdominal hysterectomy [8]. It is supported by the observation that in the literature we found that the mean time taken to perform LAVH had a very wide range with a minimum of 77 minutes [9] to 179.8 minutes [6].

## 5. Conclusion

This study showed that LAVH had a disadvantage of longer operation time but had a definitive advantage of less blood loss and less postoperative pain. The skill of laparoscopy though has a learning curve but can be mastered over time, which will lead to combating the one and only negative issue of greater operative time.

## Figures and Tables

**Table 1:** Demographic characteristics of the studied population.

Variables	LAVH *n* = 17	Abdominal hysterectomy *n* = 20	*P* value
Age (years)*			
Mean ± SD	43.2 ± 5.86	49.8 ± 8.59	0.010
Parity*			
Median (IQR)	2 (0–5)	2 (0–5)	0.619
Nulliparous	1	2	
Previous pelvic surgeries** (%)	9 (53)	9 (45)	0.193
BMI (kg/m^2^)*			
Mean ± SD	24.8 ± 5.86	25.9 ± 5.86	0.473
Comorbidities			
Hypertension (%)	2 (11.7)	8 (40)	
Diabetes mellitus	1 (5.8)	3 (15)	
Thyroid disorder	3 (17.6)	3 (15)	
Bronchial asthma	0	2 (10)	

*Independent sample *t*-test; **Mann-Whitney test.

**Table 2:** Intraoperative characteristics.

Characteristics	LAVH *n* = 17	Abdominal hysterectomy *n* = 20	*P* value
Operating time (min) Mean ± SD*	167.06 ± 31.97	135.25 ± 31.72	0.005
Estimated blood loss (mL) Mean ± SD**	248.24 ± 117.79	340.00 ± 119.86	0.003
Weight of specimen (g) Mean ± SD*	223.82 ± 71.6	252.00 ± 151.92	0.569

*Independent sample *t*-test; **Mann-Whitney test.

**Table 3:** Postoperative complications.

Complications	LAVH *n* = 17 (%)	Abdominal hysterectomy *n* = 20 (%)	*P* value
Febrile Morbidity*	3 (17.6)	1 (5)	0.482
Secondary hemorrhage*	3 (17.6)	1 (5)	0.482
Wound infection**	0	3 (15)	0.489

*Independent sample *t*-test; **Mann-Whitney test.
